# Infrared Matrix-Isolation and Theoretical Studies of the Reactions of Bis(benzene)chromium with Ozone

**DOI:** 10.3390/molecules29153583

**Published:** 2024-07-29

**Authors:** Roger W. Kugel, Bruce S. Ault

**Affiliations:** Department of Chemistry, University of Cincinnati, Cincinnati, OH 45221, USA; kugelrr@ucmail.uc.edu

**Keywords:** matrix isolation, organometallic complex, infrared spectroscopy, theoretical calculations, ozonolysis

## Abstract

Reactions of bis(benzene)chromium (Bz_2_Cr) and ozone (O_3_) were studied using low-temperature argon matrix-isolation infrared spectroscopy with supporting DFT calculations. When Bz_2_Cr and O_3_ were co-deposited, they reacted upon matrix deposition to produce two new prominent peaks in the infrared spectrum at $431\;{cm}^{-1}$ and $792\;{cm}^{-1}$. These peaks increased upon annealing the matrix to 35 K and decreased upon UV irradiation at *λ* = 254 nm. The oxygen-18 and mixed oxygen-16,18 isotopic shift pattern of the peak at $792\;{cm}^{-1}$ is consistent with the antisymmetric stretch of a symmetric ozonide species. DFT calculations of many possible ozonide products of this reaction were made. The formation of a hydrogen ozonide (H_2_O_3_) best fits the original peaks and the oxygen-18 isotope shift pattern. Energy considerations lead to the conclusion that the chromium-containing product of this reaction is the coupled product benzene-chromium-biphenyl-chromium-benzene (BzCrBPCrBz). $2{Bz}_{2}Cr+O_{3}\to H_{2}O_{3}+BzCrBPCrBz,\;{∆E}_{calc}=-52.13kcal/mol$.

## 1. Introduction

Organometallic compounds have drawn interest in recent years as precursors for the vapor phase deposition of metal-containing thin films, either by chemical vapor deposition (CVD) or by atomic layer deposition (ALD). In particular, volatile metallocene and metallo-arene compounds react in the gas phase with oxidants like oxygen or ozone to produce metal oxide or pure metal thin films that find applications in the electronics industry [1,2,3,4,5,6]. In spite of their utility in industry, little is understood about the detailed mechanisms of such reactions. We undertook a study of some of these reactions using the low-temperature matrix-isolation technique in an attempt to produce and isolate possible reaction intermediates that escape detection in higher temperature kinetic studies.

Our previous work in this area involved the reactions of ozone with ferrocene [7] and of ozone with ruthenocene [8] in argon matrices. These studies revealed that virtually no thermal reaction occurred between ozone and these metallocenes in the gas phase under twin-jet deposition conditions when the reactants encountered each other only briefly in the dark. When the matrices were irradiated with low-energy, red, or near infrared light from an LED (*λ* = 625 nm or *λ* = 880 nm), however, different photochemical reactions were initiated. In the case of ferrocene, the red light photolyzed ozone to produce ground state O(^3^P) atoms that inserted into one of the cyclopentadienyl rings to form a coordinated, six-membered ring: a pyranyl anion [7]. In the case of ruthenocene, the infrared light photolyzed ozone to produce ground state O(^3^P) atoms that reacted with one of the cyclopentadiene rings to form a coordinated cyclopentadienone displacing one H atom to the ruthenium center, forming a ruthenium hydride [8]. In neither case was a reaction of ozone with the *π*-system of the ring observed to produce a primary ozonide, secondary ozonide, or Criegee intermediate typical of the ozone reactions with cyclopentene or cyclopentadiene previously observed [9,10].

In this work, we extend our research to the metallo-bisarene compound bis(benzene)chromium (Bz_2_Cr). We report here our observations of the argon matrix reactions of O_3_ with Bz_2_Cr and propose an interpretation of the results.

## 2. Results and Discussion

1. Bis(benzene)chromium (Bz_2_Cr) blank

**Figure 1:**
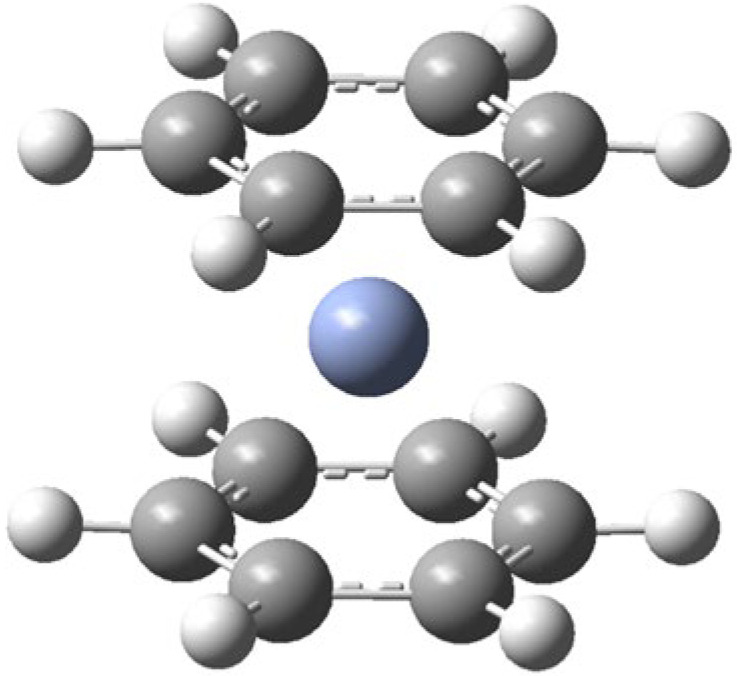
Calculated structure of Bz_2_Cr.

“Blank” spectra of bis(benzene)chromium (Bz2Cr) isolated in an argon matrix (Ar/Bz2Cr ≅200) at 15 K showed infrared absorbance peaks that agreed with those reported earlier [11] and with the DFT calculations carried out in this work. The observed and calculated absorbance peaks are listed in Table 1. The calculated structure of Bz2Cr is shown in Figure 1 below.

A few observations should be noted about the spectra summarized in Table 1. First, Boyd, Lavoie, and Gruen [11] reported an additional weak, unassigned absorbance at 432.5 ${cm}^{-1}$. This peak was neither observed in the gas-phase spectrum of Bz_2_Cr [12], nor in the argon matrix spectrum reported in this work, nor is it predicted from the DFT quantum calculations. It should be noted that Boyd et al. [11] produced Bz_2_Cr by co-deposition of benzene and atomic chromium with argon onto a liquid-helium-cooled cesium iodide window. We believe that the peak they observed at 432.5 ${cm}^{-1}$ might be due to a small amount of the less stable mono(benzene)chromium (BzCr) formed during the co-deposition of benzene and atomic chromium in the argon matrix. Secondly, the symmetry assignments given by Boyd et al. [11] for the two lowest frequency vibrations of Bz_2_Cr are reversed from those predicted by the current quantum calculations, and from those previously reported for the gas phase spectrum [13]. We believe that these assignments should be $466\;{cm}^{-1}\left(A_{2u}\right)\;and\;492\;{cm}^{-1}\left(E_{1u}\right)$, as shown in Table 1. Thirdly, the highest frequency reported in our spectrum at 3060 ${cm}^{-1}$ is actually flanked by two unresolved shoulders at about 3055 and 3066 ${cm}^{-1}$. It is possible that the highest frequency vibration predicted by the quantum calculations at 3185 ${cm}^{-1}$ is unresolved in this peak pattern. Finally, when the argon-matrix-isolated Bz_2_Cr was irradiated with ultraviolet light, new peaks appeared in the infrared spectrum indicating that a photochemical reaction had occurred. The new product, whose most prominent peak occurs at 426 cm^−1^, is likely mono(benzene)chromium, but it was not further characterized.

2. The reaction of Bz_2_Cr with O_3_ in an Ar matrix

**Figure 2:**
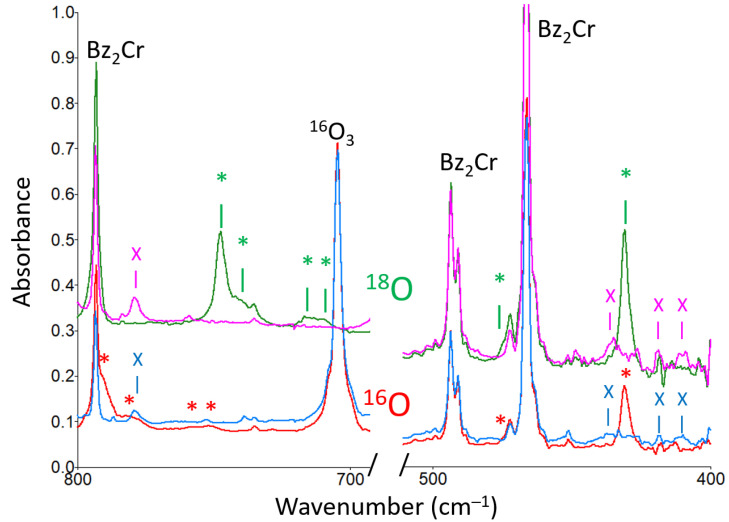
Partial infrared spectra from two experiments. Lower traces: Bz_2_Cr plus ^16^O_3_ in an argon matrix at 15 K; red trace: deposit with new peaks marked with a red asterisk * (431, 472, 752, 758, 782, and 792 cm^−1^); blue trace: after 1 h of UV irradiation with the unfiltered light from a mercury pen lamp. New peaks marked with a blue **x** (411, 419, 436, and 779 cm^−1^) are unidentified decomposition products. Upper traces: Bz_2_Cr plus ^18^O_3_ in an argon matrix at 15 K; green trace: deposit with new peaks marked with a green asterisk * (431, 472, 710, 717, 740, and 748 cm^−1^); magenta trace after 1.25 h of UV irradiation with the unfiltered light from a mercury pen lamp. New peaks marked with a magenta **x** (411, 419, 436, and 779 cm^−1^) are unidentified decomposition products. In both experiments, the new peaks appeared upon deposition, disappeared upon UV irradiation, and reappeared upon annealing the matrix to 35 K.

When gas mixtures of Bz_2_Cr plus Ar and O_3_ plus Ar were co-deposited through twin jets onto the CsI cold window, new peaks were observed in the infrared spectra of the matrices that formed. These peaks were “new” in the sense that they were not observed in similar matrices made from separate “blank” mixtures of Bz_2_Cr in Ar or of O_3_ in Ar. The most significant of these peaks, at 431 and 792 cm^−1^, increased in intensity upon warming the matrices to 35 K, and decreased in intensity upon irradiating the matrices with ultraviolet light from an unfiltered mercury pen lamp. These results suggest that O_3_ reacts with Bz_2_Cr rapidly and with little-to-no activation energy during deposition, or in the forming or annealing matrices. The results further suggest that the compound formed is readily decomposed by ultraviolet light. The new infrared peaks observed in these matrices are summarized in Table 2. Partial infrared spectra showing the major peaks are given in Figure 2.

**Figure 3:**
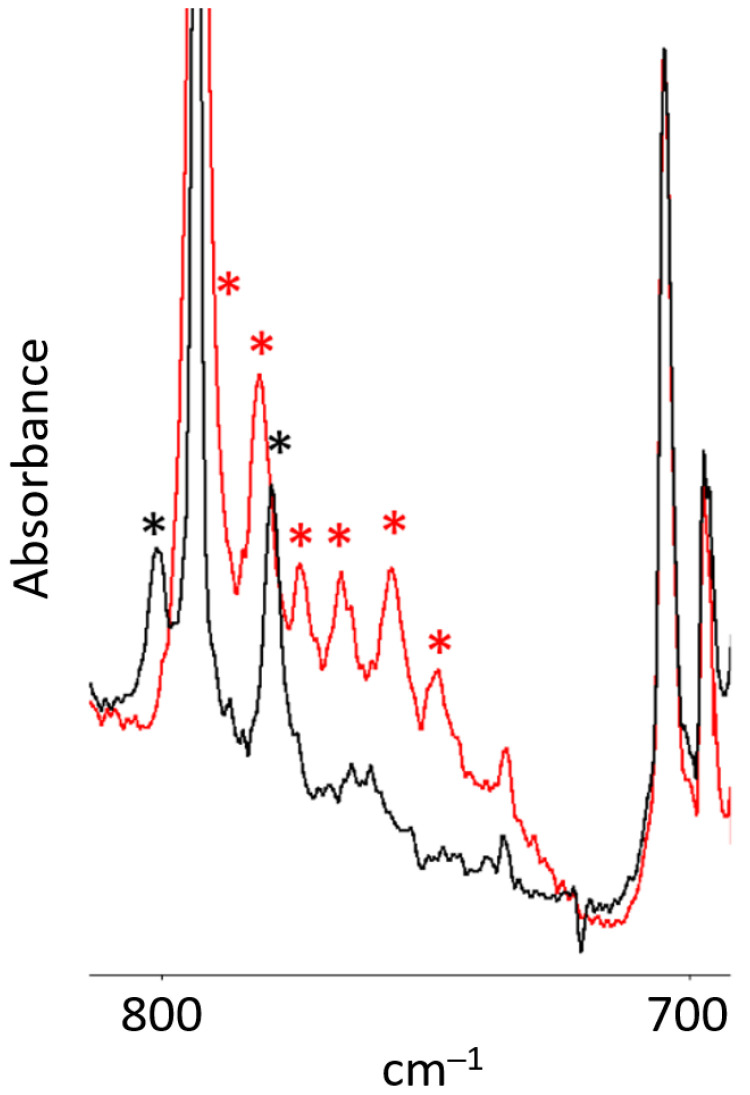
Partial infrared spectra from the mixed isotope experiment: Bz_2_Cr plus ^16, 18^O_3_ in an argon matrix at 15 K: red trace: deposit with new peaks marked with a red asterisk * (748, 756, 766, 774, 782, and 792 cm^−1^); black trace: deposit after 1.0 h of irradiation at 254 nm with the unfiltered light from a mercury pen lamp. The isotopomer peaks have disappeared and two new peaks that appeared with UV irradiation are marked with a black asterisk * (779 and 801 cm^−1^).

The most intense peak at 431 cm^−1^ in the infrared spectrum of the new species shows no significant oxygen-18 isotope shift. However, the second-most intense peak at 792 cm^−1^ shows an exceptionally large oxygen-18 isotope shift of 44 cm^−1^. This large shift suggests that this vibrational mode involves significant motion of one or more oxygen atoms. To explore this isotope shift, a mixed isotope experiment was carried out with scrambled ^16,18^O_3_ ozone. The results of this experiment revealed six isotopomer peaks between 748 and 792 cm^−1^. These peaks and their relative intensities are listed in Table 3 and shown in Figure 3. The peak pattern shown by these peaks is suggestive of the antisymmetric stretch of a symmetric ozonide moiety. The pattern shows the second and fifth peaks in the isotopic progression are more intense than the third and fourth peaks, indicating that they correspond to the following isotope sequences: 16, 16, 18 and 16, 18, 18, which would be twice as intense for the antisymmetric stretch of a symmetric ozonide.

Another interesting feature of the intense infrared absorption of the new species at 792 cm^−1^ is that it has three less-intense “satellite” peaks associated with it: a shoulder at 782 cm^−1^ and (barely resolved) peaks at 758 and 752 cm^−1^. This entire four-peak pattern appears upon deposition, increases upon annealing the matrix, and disappears upon UV irradiation. Furthermore, all four peaks show similar large oxygen-18 isotope shifts of 41 to 44 cm^−1^, so that the entire peak pattern repeats at 748, 740, 717, and 710 cm^−1^. That the behavior of the peaks in this pattern is so similar suggests that they are due to similar molecular structures possibly perturbed by environmental or conformational effects. The fact that the spread of the peak pattern for a given oxygen isotope is so large (~40 cm^−1^) seems to rule out simple matrix site effects and suggests that the satellite peaks are caused by different isomers or conformers of the new species that is formed on deposition of the matrix.

**Figure 4:**
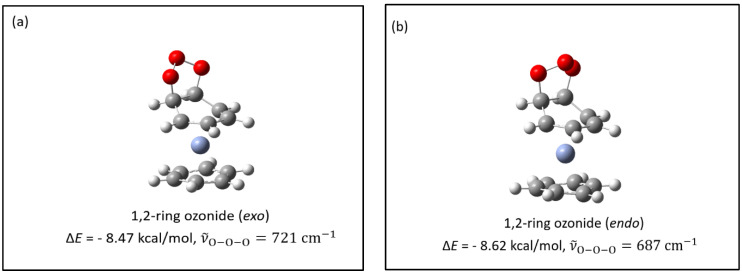

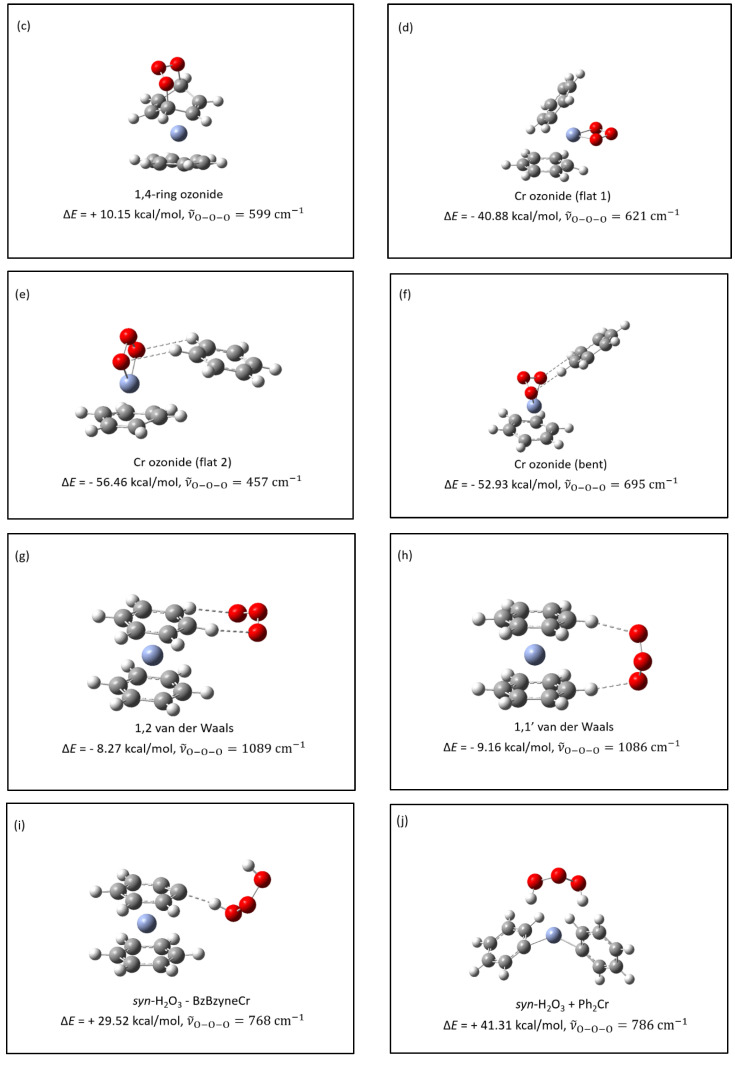

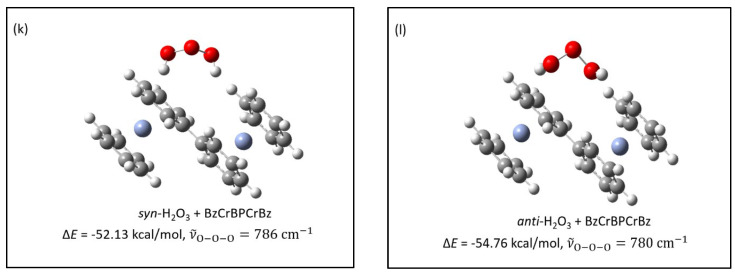
Gaussian structures with symmetric ozonides. (**a**–**j**): potential products from the reaction: ${Bz}_{2}Cr+O_{3}\to$ product; (**k**,**l**): potential products from the reaction: $2\;{Bz}_{2}Cr+O_{3}\to$ product. Δ*E* values represent the differences in energy between the structure represented and the Bz_2_Cr + O_3_ reactants; ${\tilde{\nu }}_{O-O-O}$ represents the antisymmetric vibrational frequency of the ozonide product. Reference for the calculated value for ${\tilde{\nu }}_{O-O-O}$ in ozone: 1183 cm^−1^ (239 km/mol).

Several symmetric ozonide molecules were calculated as potential candidates for the new product formed between ozone and bis(benzene)chromium in an argon matrix. Focus was placed on the calculated energy of reaction, ∆E, and the calculated frequency and intensity of the antisymmetric O-O-O stretching mode, ${\tilde{\nu }}_{asym\;O-O-O\;stretch}\;(I)$. A list of potential candidate molecules with appropriate calculated data is shown in Table 4 and the calculated Gaussian structures are shown in Figure 4.

**Figure 5:**
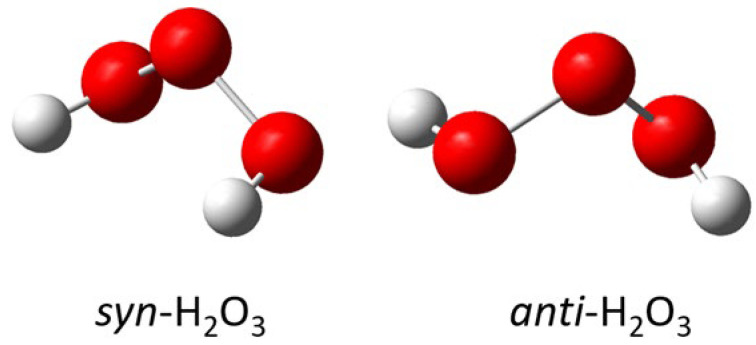
Hydrogen ozonide Gaussian calculated structures for *syn*-H_2_O_3_ and *anti*-H_2_O_3_.

**Figure 6:**
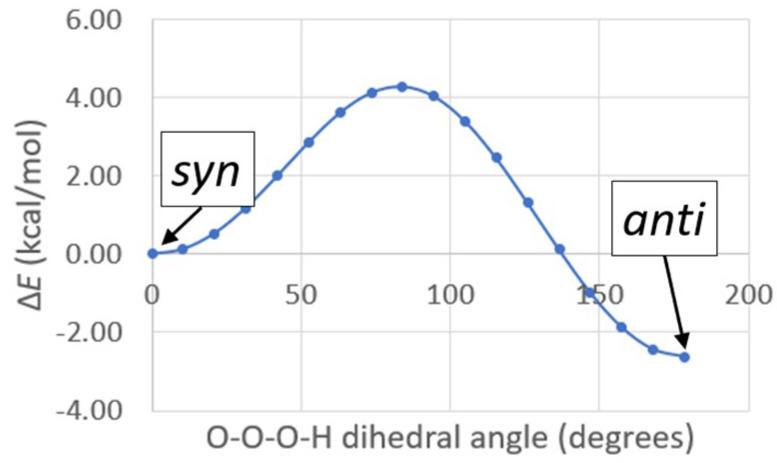
Energy diagram for *syn/anti* conversion of H_2_O_3_.

By far, of all the symmetric ozonides calculated, the one that best fits the observed infrared data is hydrogen ozonide (H_2_O_3_). The two most prominent peaks in the calculated infrared spectrum of *syn*-H_2_O_3_ are at 444 cm^−1^ ∆ν~O818=−3 cm−1 and 786 cm^−1^ ∆ν~O818=−44 cm−1, and correspond closely to the two most prominent peaks in the experimental spectrum of the new species: 431 cm^−1^ ∆ν~O818=0 cm−1 and 792 cm^−1^ ∆ν~O818=−44 cm−1. Not only is the calculated antisymmetric stretching frequency $\left({786cm}^{-1}\right)$ closest to that observed $\left({792cm}^{-1}\right)$, but the calculated oxygen-18 isotope shift $\left({-44cm}^{-1}\right)$ is also exactly the same as that observed $\left({-44cm}^{-1}\right)$. These results comparing experiment, the literature, and calculation are summarized in Table 5. Note that H_2_O_3_ has been observed in previous matrix experiments by Engdahl and Nelander, who isolated H_2_O_3_ from the argon matrix reaction of H_2_O_2_ with O(^1^D) [14]. It was also observed recently in our laboratory by Pinelo et al., who isolated H_2_O_3_ from the argon matrix reaction of ozone with 1,4-cyclohexadiene to produce benzene and hydrogen ozonide (H_2_O_3_) [15]. Hydrogen ozonide (H_2_O_3_) can be formed in *syn-* and *anti*-conformations, with the *anti*-form more stable by 2.63 kcal/mol. The *syn-* and *anti-* calculated Gaussian structures for H_2_O_3_ are shown in Figure 5. The *syn/anti* calculated energy diagram for H_2_O_3_ is shown in Figure 6.

**Figure 7:**
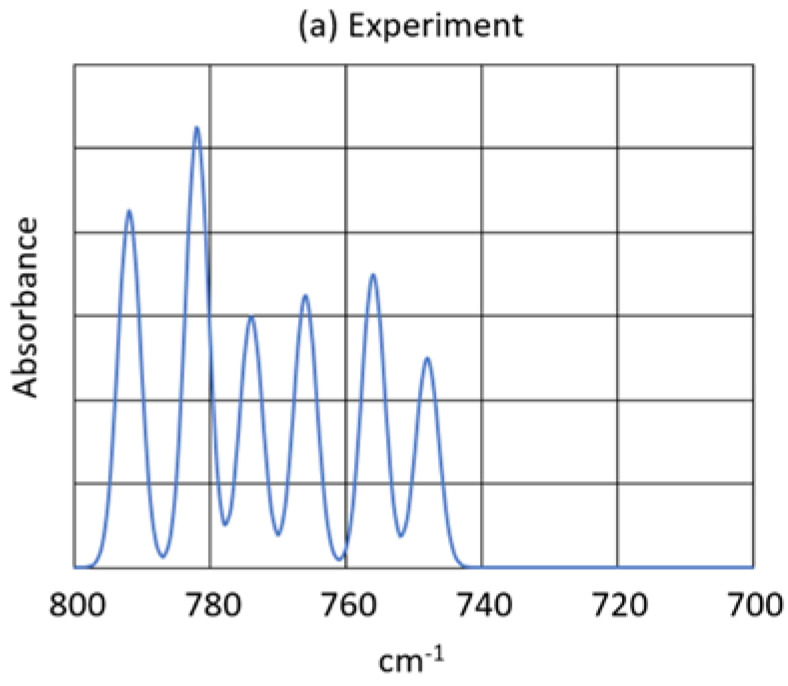

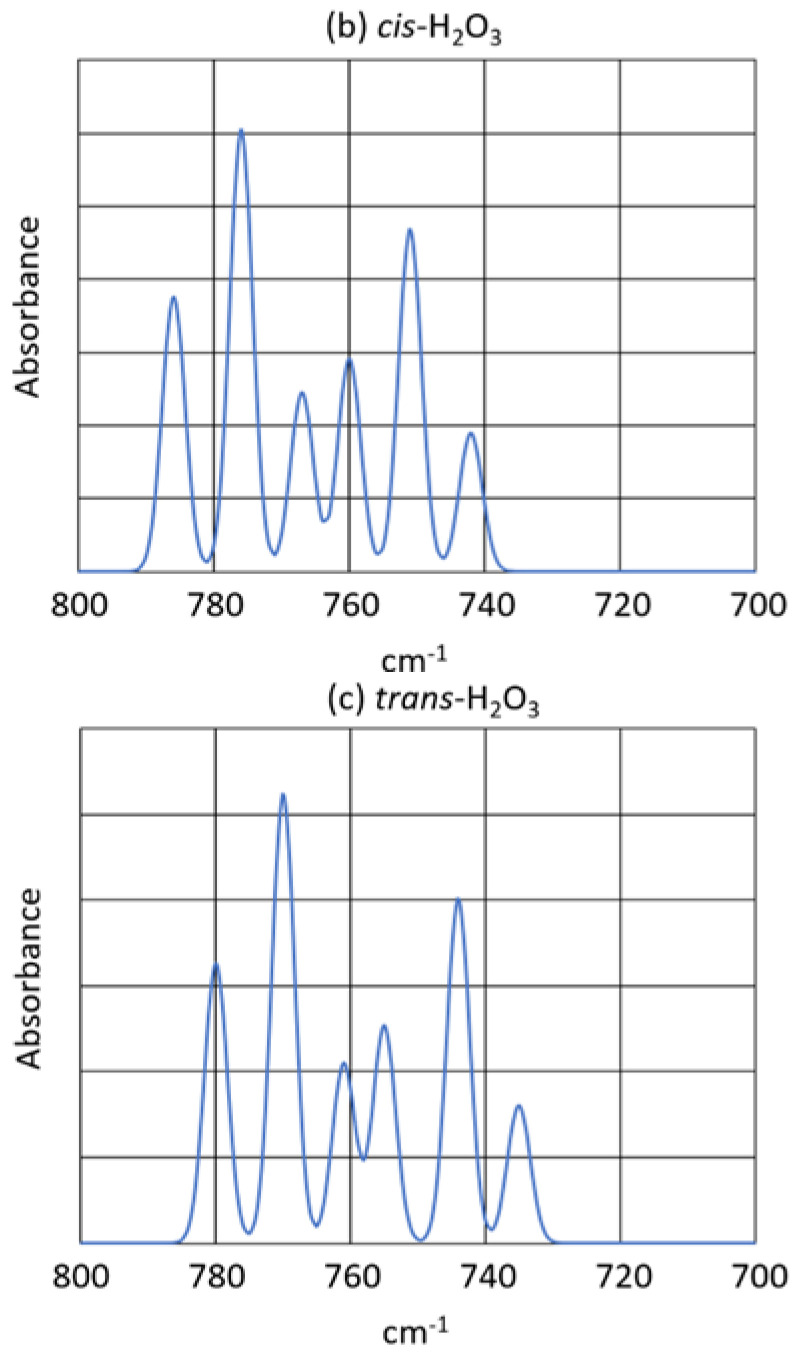
Isotopomer infrared peaks. (**a**) Experiment: Bz_2_Cr + ^16,18^O_3_. The experimental spectrum was baseline-corrected with the measured intensities and wavenumbers. (**b**) Calculated: *syn*-H_2_O_3_. (**c**) Calculated: *anti*-H_2_O_3_. The calculated spectra used the calculated wavenumbers and intensities and assumed 45% oxygen-18 in the ^16,18^O_3_ mixture.

Furthermore, the peak pattern observed in the scrambled, mixed isotope experiment (See Figure 3) is almost exactly reproduced by the calculated peaks of all six of the H_2_O_3_ isotopomers. Figure 7 shows the observed and calculated peak patterns of the ^16,18^O isotopomers of H_2_O_3_. Note that in Figure 7 the observed peaks in the experimental spectrum are recalculated for baseline correction. The isotopomer peak assignments are given in Table 6.

The peak patterns calculated for the *syn*- and *anti*-H_2_O_3_ are very similar to that observed in the Bz_2_Cr + ^16,18^O_3_ matrix experiment. The entire pattern is slightly shifted to lower frequencies by 6 ${cm}^{-1}$ for the *syn*-isomer and by 12 ${cm}^{-1}$ for the *anti*-isomer. Also, the individual oxygen-18 isotope shifts for the individual *syn*- and *anti*-H_2_O_3_ isotopomers are nearly identical to all the shifts observed in the experiment. Next, notice in the peak assignments that the 668/866 isotopomers and the 688/886 isotopomers are identical because H_2_O_3_ is a symmetrical ozonide. Finally, the isotope shift for the 686 isotopomer is greater than that for the 868 isotopomer because in the antisymmetric stretch of the O-O-O group, the middle O atom moves a greater distance than do the other two. Thus, the isotope shift for one O atom in the middle is greater than that for two O atoms on the ends of the O-O-O group. Such would not be the case for the symmetric O-O-O stretch.

The formation of hydrogen ozonide, (H_2_O_3_) begs the following questions: Where do the H atoms come from? And what is the mechanism of this formation reaction? Calculations show that O_3_ interacts strongly with hydrogen atoms of the benzene rings of Bz_2_Cr. These intermolecular interactions are shown in Figure 4g,h. In Figure 4g, the terminal O atoms of ozone are simultaneously interacting with two H atoms on the same benzene ring of Bz_2_Cr. This calculated interaction energy is ∆E=−8.27 kJ/mol and the calculated antisymmetric vibrational frequency is $1089\;{cm}^{-1}$. In Figure 4h, the terminal O atoms of ozone are also simultaneously interacting with two H atoms, in this case one on each benzene ring of Bz_2_Cr. This calculated interaction energy is ∆E=−9.16 kJ/mol and the calculated antisymmetric vibrational frequency is $1086\;{cm}^{-1}$. These calculated intermolecular interaction energies are unusually large for this type of interaction, approaching the strength of a hydrogen bond for each C-H---O encounter. It should be noted that the calculated non-covalent interactions of ozone with benzene itself are no greater than ∆E=−1.65 kJ/mol total, and the ozone O atoms are not particularly attracted to the benzene H atoms. The stronger interaction of ozone with the Bz_2_Cr hydrogen atoms is understandable in terms of the fact that the Cr atom is pulling electron density from both benzene rings, leaving the H atoms with significant positive charge. As a result, we believe that co-deposition of O_3_ with Bz_2_Cr in an argon matrix will result in a significant number of Bz_2_Cr---O_3_ van der Waals complexes forming in the matrix. It should be noted that the strong, hydrogen-bond-like interactions between the oxygen atoms of ozone and the hydrogen atoms of Bz_2_Cr are consistent with reports of similar interactions between O atoms of water and C-H bonds of Bz_2_Cr^+^ ions in crystals of hydrated salts from X-ray diffraction data [16]. In fact, the C-H---O interactions in the Bz_2_Cr+ crystals are so strong that these researchers conclude the following:

“In summary this study provides evidence that hydrogen bonds (both of the conventional OH-O and controversial CH-O types) afford a pattern of interactions in common between organics, organometallics, and water that can be utilized to engineer crystalline materials on the basis of the complementarity between donors and acceptors”.

If the O_3_ moiety of the Bz_2_Cr---O_3_ complex were able to abstract two hydrogen atoms from Bz_2_Cr, forming H_2_O_3_ + BzBzyneCr, it probably would. Unfortunately, that reaction is energetically unfavorable, as is the formation of Ph_2_Cr with H_2_O_3_. Figure 4i,j show these structures and their calculated energies as ∆E=+29.52 kJ/mol and ∆E=+41.31 kJ/mol, respectively, relative to the Bz_2_Cr + O_3_ starting materials.

**Figure 8:**
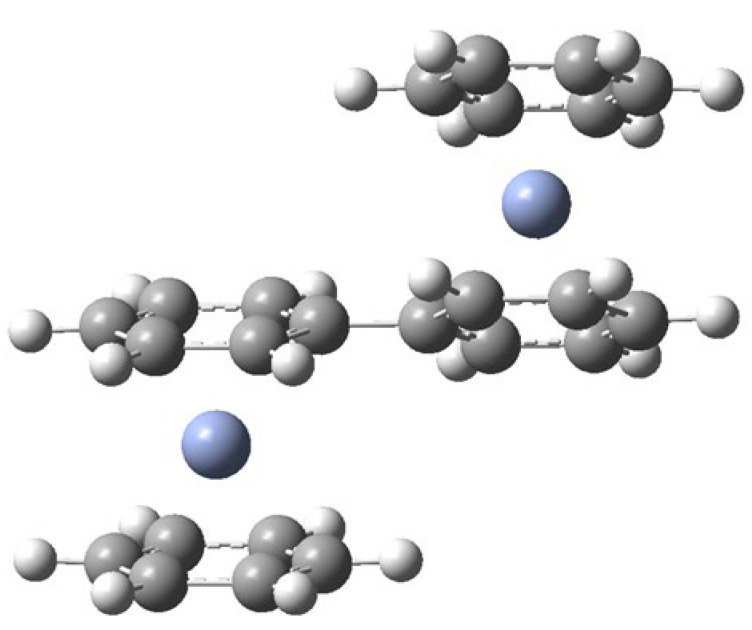
Calculated structure for BzCrBPCrBz. The calculated angle between the biphenyl rings is 27°, significantly less than that calculated for biphenyl itself (41°).

**Scheme 1:**
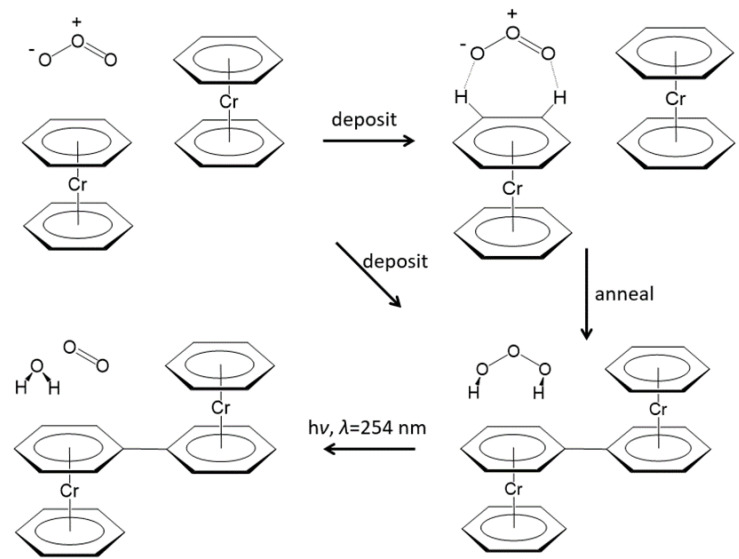
O_3_ interacts and reacts with Bz_2_Cr upon deposition and annealing of the argon matrix. H_2_O_3_ and coordinated biphenyl form during deposition and annealing. H_2_O_3_ is decomposed by UV photolysis at *λ* = 254 nm.

The Bz_2_Cr---O_3_ complexes and the uncomplexed O_3_ and Bz_2_Cr species will be fairly isolated at the beginning of the deposition. However, as deposition proceeds over a 20–24-h period, the pressures of Ar and O_3_ gases decrease and so do their deposition rates. Bz_2_Cr, on the other hand, is fed into the depositing Ar stream by its constant vapor pressure at 85 ℃. So, the relative proportion of Bz_2_Cr in the matrix increases toward the end of deposition. As a result, formation of the Bz_2_Cr---O_3_ complexes will be more numerous toward the end of the deposition process. In addition, the likelihood of these complexes encountering another molecule of Bz_2_Cr will also increase. Consequently, during late-term deposition or during annealing to 35 K, three-way encounters like Bz_2_Cr---O_3_ + Bz_2_Cr will more likely occur. These encounters of non-isolated molecules in the matrix could conceivably lead to a coupling reaction between the Bz_2_Cr species, splitting out H_2_O_3_ and probably going through a BzBzyneCr-like intermediate. This coupling would result in a coupled benzene-chromium-biphenyl-chromium-benzene (BzCrBPCrBz) product, where BP = biphenyl. The energy of this overall reaction is very favorable at ∆E=−52.13 kJ/mol for the *syn*-H_2_O_3_ and ∆E=−54.76 kJ/mol for the *anti*-H_2_O_3_ product. The calculated energies and structures are shown in Figure 4k,l. There is evidence in the literature for the formation of biphenyl from the reaction of benzene with benzyne [17,18]. The calculated structure for the BzCrBPCrBz molecule is shown in Figure 8 and the proposed mechanism for this coupling reaction is shown in Scheme 1.

Given this proposed mechanism, it seems likely that the *syn*-conformer of H_2_O_3_ is preferred in the reaction, since the interaction is a simultaneous frontal attack of ozone on two hydrogen atoms of the benzene ring of Bz_2_Cr. The more stable *anti*-conformer of H_2_O_3_ would be less prevalent. This is also consistent with the experimental spectrum, since the observed “satellite” peaks are less intense and shifted to lower frequencies from the primary peak at 792 cm^−1^. Also, the major peaks in the calculated spectrum of BzCrBPCrBz are indistinguishable from (within 2 or 3 wavenumbers of) those in Bz_2_Cr.

It should be noted that biphenyl-coordinated chromium compounds were formed in very early syntheses of chromium phenyl compounds by the reaction of phenyl magnesium bromide (PhMgBr) with anhydrous chromium(III) chloride (CrCl_3_) in benzene solution [19]. Although, at the time of this early work by Hein (1918–1936) [20], it was not known that biphenyl itself formed during the reaction to produce the metallocene compound, *η*^6^-bis(biphenyl)chromium (I) bromide. In later work, the structure we propose in Figure 8 (BzCrBPCrBz) was actually produced and characterized (among many other chromium arenes) by the direct deposition of Cr(*g*) with benzene(*g*) and biphenyl(*g*) on cold surfaces [21].

## 3. Materials and Methods

Oxygen (O_2_, 99.999%) and argon (Ar, 99.999%) gases were supplied by Wright Brothers, Inc., Cincinnati, OH, USA. Oxygen-18 isotopically enriched oxygen (^18^O_2_, 99-atom percent ^18^O) was supplied by Sigma-Aldrich Chemical Co., St. Louis, MO, USA. Ozone (O_3_ or ^18^O_3_) was produced using a high-frequency Tesla coil discharge through oxygen gas (O_2_ or ^18^O_2_) while being condensed with liquid nitrogen. To produce a statistically scrambled mix of ^16,18^O_3_ isotopomers, a 50–50 mixture of O_2_ and ^18^O_2_ was discharged. To minimize the ozone explosion hazard, the pressure of ozone gas at room temperature was never allowed to exceed 5.0 in. of Hg (127 torr). A stock supply of bis(benzene)chromium (Bz_2_Cr) was obtained from Strem Chemicals, Inc., Newburyport, MA, USA, and purified by sublimation. Gas mixtures of ozone with argon (at mole ratios of approximately Ar/O_3_ = 200) were prepared by standard manometric techniques. Ar and O_3_ were premixed, and the Ar/Bz_2_Cr mixtures were prepared by the sublimation of the bis(benzene)chromium at ∼85 °C into a flowing stream of pure argon during deposition. Both mixtures were deposited using stainless steel needle valves onto a cryogenically cooled CsI window held at 10−15 K with a closed-cycle helium cryostat (CTI Cryogenics, now from Brooks Automation, Inc., Chelmsford, MA, USA). Matrix formation took place over 20−24 h at an average argon deposition rate of 2–3 mmol/h.

The infrared spectra of the argon matrices were scanned using FT-IR spectroscopy (PerkinElmer Spectrum One, Waltham, MA, USA) at $1\;{cm}^{-1}$ resolution from 4000 to 400 ${cm}^{-1}$. After deposition, the matrices were subsequently either irradiated or annealed to observe by infrared spectroscopy any chemical changes that may have occurred. Irradiation was performed with ultraviolet light from a mercury pen lamp (Pen-Ray PS-1, UVP Cambridge, Cambridge, UK) whose primary output is the Hg line at a wavelength of *λ* = 254 nm. Annealing was accomplished with a small resistance heater attached to the brass mounting for the CsI cold window.

Quantum chemistry calculations (geometry optimizations, total energies, and infrared vibrational frequencies) were carried out with the Gaussian 09 suite of programs (Revision C.01) [22] using density functional theory (DFT) and the B3LYP functionals with the 6-311G++(d,2p) basis set. Gaussian calculations were carried out remotely at the Ohio Supercomputer Center (OSC) in Columbus, OH, USA.

## 4. Conclusions

1. Bis(benzene)chromium (Bz_2_Cr) was isolated in an Ar matrix from the vapor pressure of Bz_2_Cr(*s*) at 85 °C fed into a stream of argon gas.
2. The infrared spectrum of the resulting matrix-isolated Bz_2_Cr agrees with the infrared spectrum of matrix-isolated Bz_2_Cr produced by co-deposition of C_6_H_6_(*g*) and Cr(*g*) with Ar(*g*) [11].
3. When bis(benzene)chromium (Bz_2_Cr) was co-deposited in the dark with ozone (O_3_), a new product was formed upon deposition that increased upon annealing to 35 K, and was destroyed by UV irradiation at 254 nm.
4. The new product showed two strong bands in the infrared spectrum, at $431\;{cm}^{-1}$ and at $792\;{cm}^{-1}$, as well as some minor peaks. The $431\;{cm}^{-1}$ peak showed virtually no oxygen-18 isotope shift, but the $792\;{cm}^{-1}$ peak showed an exceptionally large oxygen-18 isotope shift consistent with the antisymmetric O-O-O stretch of a symmetric ozonide product.
5. The structures of several possible symmetric ozonide products of the reaction between O_3_ and Bz_2_Cr were calculated. The best fit of the antisymmetric stretch and its oxygen-18 isotope shifts was shown by hydrogen ozonide (H_2_O_3_) and its oxygen-18 isotopomers.
6. The formation of H_2_O_3_ in this reaction is undoubtedly facilitated by the unusually strong intermolecular interactions of ozone with the Bz_2_Cr hydrogen atoms, which have calculated interaction energies of −8.27 to −9.16 kcal/mol, rivaling those of traditional hydrogen bonding.
7. H_2_O_3_ can be formed in an energetically favorable process if the hydrogen-deficient benzene-benzyne-Cr (BzByCr) couples with another Bz_2_Cr molecule and rearranges to benzene-Cr-biphenyl-Cr-benzene (BzCrBPCrBz), which was previously observed [21].

## Figures and Tables

**Table 1:** Observed and calculated infrared absorbance peaks for bis(benzene)chromium (Bz_2_Cr) isolated in an argon matrix at 15 K.

Experiment ^a^		Literature ^b^		Calculations ^a^		
Bz_2_Cr Vaporized at 85 C and Co-Deposited with Argon at 15 K		$Cr+{2C}_{6}H_{6}\overset{Armatrix}{\to }{Bz}_{2}Cr$ Bz_2_Cr Formed in the Ar Matrix on Deposition		DFT/B3LYP 6-311G++(d,2p)		
$\tilde{\nu }\;({cm}^{-1})$	*A* (a.u.) ^c^	$\tilde{\nu }\;({cm}^{-1})$	*A* (a.u.)	$\tilde{\nu }\;({cm}^{-1})$	$I\left(km/mol\right)$ ^d^	Assignment
467	0.65	466	0.81	435	177 d	$A_{2u}$
491 494	0.16 0.20	492	0.23	478 478	42 42	$E_{1u}\;E_{1u}$
793	0.22	793	0.15	798	58	$A_{2u}$
861	0.04	862	0.02	876 876	7 7	$E_{1u}\;E_{1u}$
974	0.10	974	0.13	986	25	$A_{2u}$
1003	0.03	1001	0.03	1021 1021	9 9	$E_{1u}\;E_{1u}$
1433	0.07	1433	0.06	1466 1466	2 2	$E_{1u}\;E_{1u}$
3060	0.19	3062	0.11	3179 3179	83 83	$E_{1u}\;E_{1u}$
				3185	18	$A_{2u}$

^a^ This work; ^b^ Reference [11]. ^c^ *A* (a.u.) is absorbance in absorbance units. ^d^ *I* (km/mol) is calculated intensity in kilometers per mole.

**Table 2:** New infrared peaks observed in the twin-jet co-deposition of gas mixtures: Ar/Bz_2_Cr ≅ 200 and Ar/O_3_ ≅ 200 onto a CsI window at 15 K.

New Peaks Observed upon Deposition ^a^ of Bz_2_Cr and O_3_ in Solid Ar at 15 K		
$\;\tilde{\nu }\;({cm}^{-1})\;$	*A*^b^ (a.u.)	$\;{\Delta \tilde{\nu }}_{O^{18}}\;({cm}^{-1}{)}^{c}\;$
431	0.16	0
472 ^d^	0.03	0
622	0.02	−35
752	0.02	−42
758	0.02	−41
782	0.03	−42
792	0.12	−44
865 ^d^	0.02	−23
890 ^d^	0.03	−21
1007 ^d^	0.03	-
1431	0.03	−2

^a^ Note that all the peaks listed in this table appeared upon deposition, increased upon annealing to 35 K, and decreased upon UV irradiation at 254 nm. ^b^ The intensities listed were those measured after 18 h of deposition. **^c^**∆ν_O_(O-18) is the oxygen-18 isotope shift in wavenumbers (cm^−1^). ^d^ The peaks found at 472, 865, 890, and 1007 cm^−1^ are due to trace amounts of unassigned byproducts that remain unknown.

**Table 3:** New isotopomer infrared peaks observed in the twin-jet co-deposition of gas mixtures: Ar/Bz_2_Cr ≅ 200 and Ar/^16,18^O_3_ ≅ 200 onto a CsI window at 15 K. Isotopomer peaks for the ^16,18^O_3_ scrambled, mixed isotope experiment.

Isotopomer Peaks for the ^16,18^O_3_ Scrambled Mixed Isotope Experiment	
$\;\tilde{\nu }\;({cm}^{-1})$	*A* (a.u.)
748	0.040
756	0.056
766	0.052
774	0.048
782	0.084
792	? ^a^

^a^ Note that the intensity of the peak at 792 cm^−1^ could not be accurately determined due to its proximity to the intense parent peak for Bz_2_Cr at 793 cm^−1^.

**Table 4:** Calculated symmetric ozonide molecules as potential products of the reaction between Bz_2_Cr and O_3_ in an argon matrix. The calculated values of ∆E are relative to the separated molecules. The frequencies listed are calculated frequencies for the antisymmetric O-O-O stretch. The calculated frequency and intensity values for ozone are included at the end of the list for reference.

	Symmetric Ozonide	∆E	${\tilde{\nu }}_{\;asym\;O-O-O\;stretch}\;(I)$
	${\mathrm{Bz}}_{2}\mathrm{Cr}+O_{3}\;\to$	(kcal/mol)	(cm^−1^) (km/mol)
(a)	1,2-ring ozonide (*exo*)	−8.47	721 (14)
(b)	1,2-ring ozonide (*endo*)	−8.62	687 (20)
(c)	1,4-ring ozonide	+10.15	599 (14)
(d)	Cr-ozonide (flat 1)	−40.88	621 (26)
(e)	Cr-ozonide (flat 2)	−56.46	457 (6)
(f)	Cr-ozonide (bent)	−52.93	695 (33)
(g)	1,2 van der Waals	−8.27	1089 (168)
(h)	1,1′ van der Waals	−9.16	1086 (155)
(i)	*syn*-H_2_O_3_ + BzByCr	+29.52	786 (113)
(j)	*syn*-H_2_O_3_ + Ph_2_Cr	+41.31	786 (113)
	**2 Bz_2_Cr + O_3_** →		
(k)	*syn*-H_2_O_3_ + BzCrBPCrBz	−52.13	786 (113)
	**2 Bz_2_Cr + O_3_** →		
(l)	*anti*-H_2_O_3_ + BzCrBPCrBz	−54.76	780 (98)
(m)	Ozone, O_3_^a^	---	1183 (239)

^a^ Note that the calculated frequency of ozone is included for reference.

**Table 5:** The new peaks observed in the argon matrix reaction of O_3_ + Bz_2_Cr compared with those previously observed for H_2_O_3_ and with those calculated for H_2_O_3_.

Experiment ^a^			Literature ^b^	Calculations ^a^		
New Peaks Observed upon Deposition of Bz_2_Cr and O_3_ in Solid Ar at 15 K			$O_{3}+H_{2}O_{2}\overset{h\nu }{\to }O_{2}+H_{2}O_{3}$	$H_{2}O_{3}$ DFT/B3LYP 6-311G++(d,2p)		
$\;\tilde{\nu }\;({cm}^{-1})$	*A* (a.u.)	Δν~O818(cm−1)	$\;\tilde{\nu }\;({cm}^{-1})$	$\;\tilde{\nu }\;({cm}^{-1})$	$\;I\;\left(km/mol\right)$	Δν~O818(cm−1)
431	0.14	0	387	444	118	−3
622	0.02	−35	509	514	9	−29
792	0.10	−44	776	786	113	−44
1431	0.03	−2	1359	1377	45	−6
-	-	-	3530	3731	36	−13

^a^ This work. ^b^ Reference [14].

**Table 6:** Isotopomer peak assignments for the antisymmetric stretch of H_2_O_3_. In the assignment column, 666 represents H-^16^O-^16^O-^16^O-H, 868 represents H-^18^O-^16^O-^18^O-H, etc.

Experiment		*syn*-H_2_O_3_		*anti*-H_2_O_3_		Assignment
$\tilde{\nu }$	Δν~O818	$\tilde{\nu }$	Δν~O818	$\tilde{\nu }$	Δν~O818	6 Represents ^16^O
$\left({cm}^{-1}\right)$	$\left({cm}^{-1}\right)$	$\left({cm}^{-1}\right)$	$\left({cm}^{-1}\right)$	$\left({cm}^{-1}\right)$	$\left({cm}^{-1}\right)$	8 Represents ^18^O
792	0	786	0	780	0	666
782	10	776	10	770	10	668 and 866
774	18	767	19	761	19	868
766	26	760	26	755	25	686
756	36	751	35	744	36	688 and 886
748	44	742	44	735	45	888

## Data Availability

The original contributions presented in the study are included in the article, further inquiries can be directed to the corresponding author.
